# Delayed Diagnosis of Anti-Lgi1 Autoimmune Encephalitis With Catatonia in a Patient Initiallytreated For Primary Psychiatric Disorder: Case Report

**DOI:** 10.1192/j.eurpsy.2026.11064

**Published:** 2026-07-31

**Authors:** F. J. Cruz Aviña, O. Meneses Luna, A. Salazar Rodriguez, M. G. Ochoa Madrigal, D. N. Moya Sanchez, E. F. Espinoza de Avila, L. K. Sánchez Hernandez, J. Mendez

**Affiliations:** Liason Psychiatry, ISSSTE, Mexico City, Mexico

## Abstract

**Introduction:**

Anti-LGI1 autoimmune encephalitis can initially present with affective, psychotic, or behavioral symptoms, leadingto misdiagnosis as a primary psychiatric condition. Catatonia associated with this entity is rare and oftenunderrecognized. We report a case of delayed diagnosis, where catatonia emerged in the context of a progressiveneuropsychiatric syndrome later confirmed as anti-LGI1 autoimmune encephalitis.

**Objectives:**

We report a case of delayed diagnosis, where catatonia emerged in the context of a progressiveneuropsychiatric syndrome later confirmed as anti-LGI1 autoimmune encephalitis.

**Methods:**

A 49-year-old male presented with an 8-month evolution of neuropsychiatric symptoms, including panic attacks,hallucinations, disorganized behavior, and faciobrachial dystonic seizures. He received multiple psychiatrictreatments with no sustained improvement. In early 2025, neurology evaluation included a brain MRI showingcortical atrophy and temporal FLAIR hyperintensities; EEG showed diffuse delta slowing and temporal sharp waves.CSF analysis revealed elevated protein levels without pleocytosis. Serology confirmed anti-LGI1 antibodies. InMarch 2025, psychiatry assessed catatonia using the Bush-Francis Catatonia Rating Scale (BFCRS), scoring 10 onscreening and 18 on severity. Treatment with lorazepam and quetiapine led to significant clinical improvement.Rituximab was later administered with favorable neurological stabilization

**Results:**

The patient exhibited catatonic features including fixed gaze, rigidity, echolalia, automatic obedience, stereotypies,and disorganized speech. Marked clinical improvement followed immunotherapy and benzodiazepines.

**Image 1:**
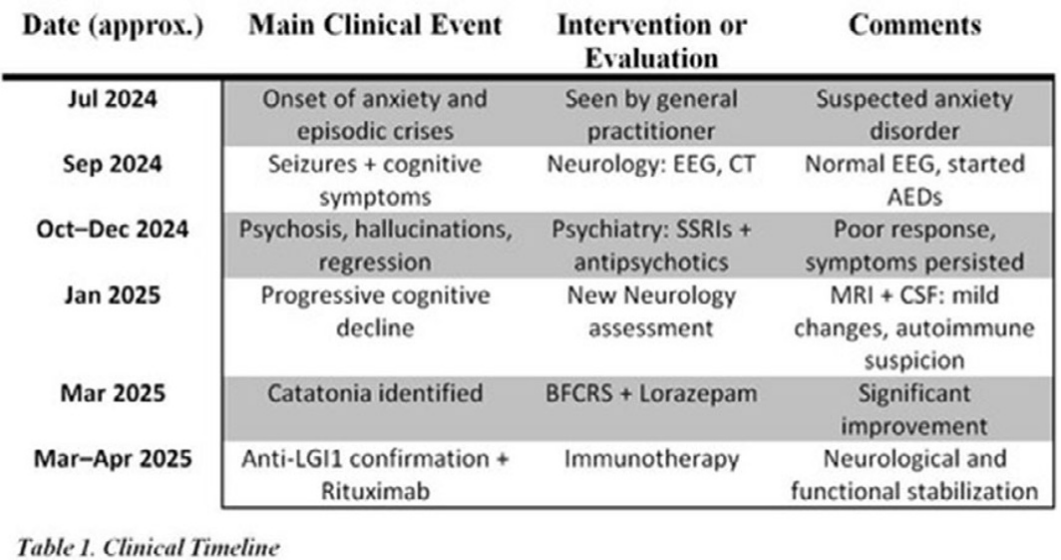
Image 1: Long description.

**Image 2:**
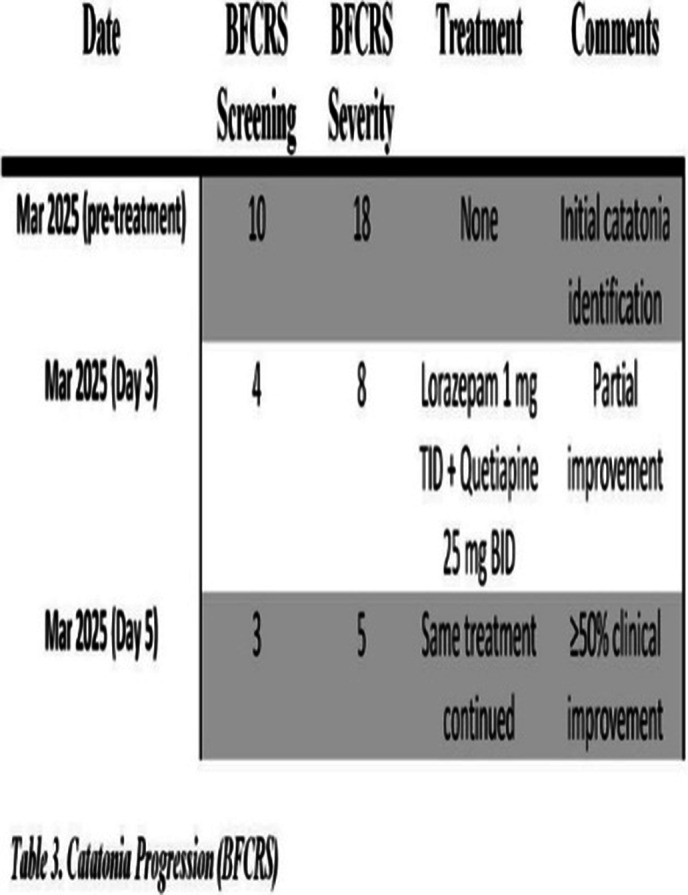
Image 2: Long description.

**Image 3:**
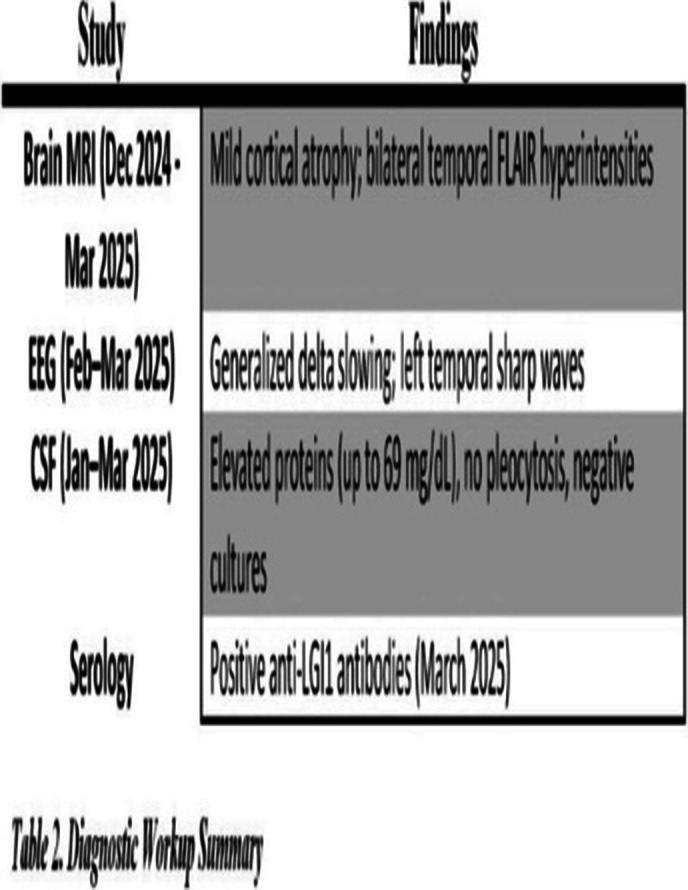
Image 3: Long description.

**Conclusions:**

This case highlights the importance of considering autoimmune encephalitis in patients with atypical psychiatricsymptoms and poor response to psychotropics. Early neurological evaluation and systematic screening forcatatonia can significantly improve diagnostic accuracy and outcomes. Multidisciplinary collaboration is crucial foridentifying catatonia as a possible manifestation of underlying neuroinflammatory disorders.

**Disclosure of Interest:**

None Declared

## Long descriptions

### Image 1: Long description

The table consists of four columns: Date (approx.), Main Clinical Event, Intervention or Evaluation, and Comments.

* July 2024: Onset of anxiety and episodic crises. Seen by general practitioner. Suspected anxiety disorder.

* September 2024: Seizures plus cognitive symptoms. Neurology: E E G, C T. Normal E E G, started A E Ds.

* October to December 2024: Psychosis, hallucinations, regression. Psychiatry: S S R Is plus antipsychotics. Poor response, symptoms persisted.

* January 2025: Progressive cognitive decline. New Neurology assessment. M R I plus C S F: mild changes, autoimmune suspicion.

* March 2025: Catatonia identified. B F C R S plus Lorazepam. Significant improvement.

* March to April 2025: Anti-L G I 1 confirmation plus Rituximab. Immunotherapy. Neurological and functional stabilization.

Navigate back to Image 1:.

### Image 2: Long description

The table consists of five columns: Date, B F C R S Screening, B F C R S Severity, Treatment, and Comments.

* Row 1 (Header): Date, B F C R S Screening, B F C R S Severity, Treatment, Comments.

* Row 2 (Pre-treatment): Date is Mar 2025 (pre-treatment). B F C R S Screening is 10. B F C R S Severity is 18. Treatment is None. Comments state Initial catatonia identification.

* Row 3 (Day 3): Date is Mar 2025 (Day 3). B F C R S Screening is 4. B F C R S Severity is 8. Treatment is Lorazepam 1 mg T I D plus Quetiapine 25 mg B I D. Comments state Partial improvement.

* Row 4 (Day 5): Date is Mar 2025 (Day 5). B F C R S Screening is 3. B F C R S Severity is 5. Treatment is Same treatment continued. Comments state greater than or equal to 50% clinical improvement.

A caption below the table reads Table 3. Catatonia Progression (B F C R S).

Navigate back to Image 2:.

### Image 3: Long description

The table consists of two columns: Study and Findings.

* Row 1: Brain M R I (Dec 2024 - Mar 2025). Findings: Mild cortical atrophy; bilateral temporal F L A I R hyperintensities.

* Row 2: E E G (Feb-Mar 2025). Findings: Generalized delta slowing; left temporal sharp waves.

* Row 3: C S F (Jan-Mar 2025). Findings: Elevated proteins (up to 69 mg/dL), no pleocytosis, negative cultures.

* Row 4: Serology. Findings: Positive anti-L G I 1 antibodies (March 2025).

The table is labeled at the bottom as Table 2. Diagnostic Workup Summary.

Navigate back to Image 3:.

